# BMAL2 promotes eCIRP-induced macrophage endotoxin tolerance

**DOI:** 10.3389/fimmu.2024.1426682

**Published:** 2024-06-13

**Authors:** Mian Zhou, Monowar Aziz, Jingsong Li, Alok Jha, Gaifeng Ma, Atsushi Murao, Ping Wang

**Affiliations:** ^1^ Center for Immunology and Inflammation, The Feinstein Institutes for Medical Research, Manhasset, NY, United States; ^2^ Departments of Surgery and Molecular Medicine, Zucker School of Medicine at Hofstra/Northwell, Manhasset, NY, United States

**Keywords:** eCIRP, macrophages, circadian, BMAL2, PD-L1, immune tolerance

## Abstract

**Background:**

The disruption of the circadian clock is associated with inflammatory and immunological disorders. BMAL2, a critical circadian protein, forms a dimer with CLOCK, activating transcription. Extracellular cold-inducible RNA-binding protein (eCIRP), released during sepsis, can induce macrophage endotoxin tolerance. We hypothesized that eCIRP induces BMAL2 expression and promotes macrophage endotoxin tolerance through triggering receptor expressed on myeloid cells-1 (TREM-1).

**Methods:**

C57BL/6 wild-type (WT) male mice were subjected to sepsis by cecal ligation and puncture (CLP). Serum levels of eCIRP 20 h post-CLP were assessed by ELISA. Peritoneal macrophages (PerM) were treated with recombinant mouse (rm) CIRP (eCIRP) at various doses for 24 h. The cells were then stimulated with LPS for 5 h. The levels of TNF-α and IL-6 in the culture supernatants were assessed by ELISA. PerM were treated with eCIRP for 24 h, and the expression of PD-L1, IL-10, STAT3, TREM-1 and circadian genes such as BMAL2, CRY1, and PER2 was assessed by qPCR. Effect of TREM-1 on eCIRP-induced PerM endotoxin tolerance and PD-L1, IL-10, and STAT3 expression was determined by qPCR using PerM from TREM-1^-/-^ mice. Circadian gene expression profiles in eCIRP-treated macrophages were determined by PCR array and confirmed by qPCR. Induction of BMAL2 activation in bone marrow-derived macrophages was performed by transfection of BMAL2 CRISPR activation plasmid. The interaction of BMAL2 in the PD-L1 promoter was determined by computational modeling and confirmed by the BIAcore assay.

**Results:**

Serum levels of eCIRP were increased in septic mice compared to sham mice. Macrophages pre-treated with eCIRP exhibited reduced TNFα and IL-6 release upon LPS challenge, indicating macrophage endotoxin tolerance. Additionally, eCIRP increased the expression of PD-L1, IL-10, and STAT3, markers of immune tolerance. Interestingly, TREM-1 deficiency reversed eCIRP-induced macrophage endotoxin tolerance and significantly decreased PD-L1, IL-10, and STAT3 expression. PCR array screening of circadian clock genes in peritoneal macrophages treated with eCIRP revealed the elevated expression of BMAL2, CRY1, and PER2. In eCIRP-treated macrophages, TREM-1 deficiency prevented the upregulation of these circadian genes. In macrophages, inducible BMAL2 expression correlated with increased PD-L1 expression. In septic human patients, blood monocytes exhibited increased expression of BMAL2 and PD-L1 in comparison to healthy subjects. Computational modeling and BIAcore assay identified a putative binding region of BMAL2 in the PD-L1 promoter, suggesting BMAL2 positively regulates PD-L1 expression in macrophages.

**Conclusion:**

eCIRP upregulates BMAL2 expression via TREM-1, leading to macrophage endotoxin tolerance in sepsis. Targeting eCIRP to maintain circadian rhythm may correct endotoxin tolerance and enhance host resistance to bacterial infection.

## Introduction

Macrophage endotoxin tolerance is manifested by a reduced responsiveness to lipopolysaccharides (LPS) (1). Immune tolerance increases the risks of secondary infections, increasing morbidity and mortality from sepsis, trauma, and ischemia/reperfusion (I/R) injuries (2, 3). The mechanisms underlying macrophage endotoxin tolerance have primarily been studied in the context of LPS-Toll-like receptor-4 (TLR4)-mediated endotoxin tolerance or homotolerance (1). However, endotoxin tolerance can also arise through pre-exposure of macrophages to TLR2 ligands, such as lipoteichoic acid, a phenomenon referred to as cross-tolerance. Additionally, chronic exposure to tumor necrosis factor-α (TNF-α) and interleukin (IL)-1β can induce endotoxin tolerance in macrophages (1). While it has been established that damage-associated molecular patterns (DAMPs) can trigger tolerance in macrophages (4), the mechanism of DAMP-mediated endotoxin tolerance remains much less characterized.

Immune checkpoint molecules, such as program death-ligand-1 (PD-L1), play a critical role in maintaining self-tolerance and preventing uncontrolled inflammation (5). PD-L1 dampens T-cell responses and contributes to the immune-suppressive microenvironment. Tumor-infiltrating myeloid cells (TIMs), including tumor-associated macrophages (TAMs), tumor-associated neutrophils, and myeloid-derived suppressor cells, are the primary contributors to PD-L1 expression (5, 6). Recent studies indicate that PD-L1 inhibitors can shift TAMs towards an M1 status (7). PD-L1 antibody-treated macrophages show increased proliferation, survival, and activation compared to control antibody-treated macrophages (7). Macrophages from PD-L1^-/-^ mice display spontaneous proliferation and activation (8). While PD-L1 has been associated with immune-tolerant macrophages, the mechanisms underlying their heightened expression has not been investigated.

Cold-inducible RNA-binding protein (CIRP), plays a crucial role in intracellularly regulating the translation of stress response genes (9). Its expression spans various cell types, including macrophages, neutrophils, and epithelial and endothelial cells. Under conditions such as hypoxia, sepsis, and hemorrhagic shock, CIRP can be released into the extracellular space (10). Extracellular CIRP (eCIRP) has been identified as a new DAMP that contributes to inflammation and organ injury in sepsis (11). In macrophages, eCIRP triggers inflammation by binding to its receptor triggering receptor expressed on myeloid cells-1 (TREM-1) (12). While the inflammatory effects of eCIRP have been extensively studied, its potential role in promoting immune tolerance in macrophages is still not fully understood.

The circadian clock exerts influence over a myriad of physiological targets, with particular emphasis on the immune systems (13–16). This hierarchical organization features a central pacemaker located in the suprachiasmatic nucleus (SCN) of the brain, along with subordinate clocks present in nearly all peripheral tissues. Through the detection and integration of light signaling, the SCN adjusts its timing in response to external day/night cycles and subsequently imparts cues to synchronize peripheral clocks (17). At the cellular level, a molecular clock machinery is expressed, sustaining circadian oscillations in various cellular functions, including gene expression, protein translation, intracellular signaling, metabolism, and cell type-specific activities (17, 18). Circadian rhythms are driven by the circadian clock, composed of interlocked transcriptional-translational feedback loops. The circadian locomotor output cycles kaput (CLOCK) and brain and muscle aryl hydrocarbon receptor nuclear translocator-like 1 (BMAL1) transcription factors heterodimerize and bind to E-box promoter elements, initiating the gene expression of period (PER1-3) and cryptochrome (CRY1/2) (13, 19). PER/CRY protein complexes reside in the cytoplasm, relocating into the nucleus later, where they inhibit the activity of the CLOCK-BMAL1 complex (20, 21). Disruption of the circadian clock is associated with various disorders, including inflammatory, and immunological diseases (16, 22). Notably, an isotype of BMAL (BMAL2) can heterodimerize with CLOCK, forming a dimer that binds to E-box elements and activates transcription (23).

In this study, we hypothesized that eCIRP modulates BMAL2 expression, promoting macrophage endotoxin tolerance. Our current study presents evidence on the critical role of BMAL2 in macrophage immune tolerance. Specifically, BMAL2 increases the expression of PD-L1. These findings establish a direct link between the circadian clock and immune tolerance in inflammation.

## Materials and methods

### Experimental animals

C57BL/6 male mice were purchased from Charles River Laboratories (Fairfield, NJ). The TREM-1^–/–^ mice were generated by the trans-NIH Knockout Mouse Project (KOMP) and obtained from the KOMP Repository, University of California, Davis (12). All experiments utilized age-matched healthy mice aged 9-12 weeks, and only male mice were included in the study. Both male and female sex steroids have been documented to exert diverse immune-modulating functions in humoral and cell-mediated immune responses under normal and various disease conditions (24). Since males and females exhibit distinct circadian rhythms, which could potentially impact the study’s findings (25, 26), we have specifically used male mice in this study to exclude the effect of sexual dimorphism on circadian gene expression. Animals were housed in a temperature-controlled room with a 12-hour light/12-hour dark cycle and fed a standard Purina rodent chow diet. Mice were allowed to acclimate to the environment for at least 7 days before being used in experiments. All experiments were performed following the National Institutes of Health guidelines for experimental animals and were approved by the Institutional Animal Care and Use Committee (IACUC).

### Experimental model of sepsis

Wild-type male mice were subjected to sepsis induction through cecal ligation and puncture (CLP) (12). Anesthesia was administered via 2% isoflurane inhalation, followed by shaving and disinfection of the abdominal area with povidone-iodine. A 1.5-cm midline incision allowed exposure of the cecum, which was ligated with 4-0 silk suture 1 cm proximal to the distal cecal tip. Subsequently, a through-and-through puncture was performed using a 22-gauge needle, extruding a small amount of cecal content. The cecum was then repositioned in the abdominal cavity, and the incision was closed in layers. Sham-operated mice underwent laparotomy without cecal ligation or puncture. After 20 hours post-CLP, mice were euthanized through CO_2_ asphyxiation, and serum samples were collected to assess eCIRP levels.

### Recombinant mouse CIRP

Recombinant mouse CIRP (rmCIRP, i.e., eCIRP) was prepared in-house, and quality control assays were performed as described previously (11). The quality of the purified protein was assessed by Ponceau staining and Western blotting. A functional assay was performed by assessing the TNF-α levels in macrophages after treatment with purified rmCIRP. The level of endotoxin in the purified protein was measured by a limulus amebocyte lysate assay kit (Lonza, Basel, Switzerland). Only the purified protein lots that were free from endotoxin were used for experiments. We performed these quality control assays for each purified protein lot.

### Isolation of peritoneal macrophages and cell culture

Peritoneal macrophages (PerM) were isolated from adult male mice, with euthanasia performed through CO_2_ asphyxiation. Cells from the peritoneal cavity were collected via peritoneal lavage using cold Hanks’ Balanced Salt Solution (HBSS) without Ca^2+^ and Mg^2+^, supplemented with 2% FBS. Total peritoneal cells were isolated by centrifugation at 300 × g for 10 minutes at 4°C and subsequently cultured in complete RPMI 1640 medium [RPMI 1640 supplemented with 10% heat-inactivated fetal bovine serum (FBS), 2 mM glutamine, 100 IU/ml penicillin–streptomycin, and 25 mM HEPES] at 37°C in 5% CO_2_. After 2 hours, nonadherent cells were removed, and adherent cells, predominantly macrophages, were cultured overnight before use. Immortalized mouse bone marrow-derived macrophages (iBMDMs) were procured from Applied Biological Materials (Cat# T0673; Richmond, Vancouver, Canada) and cultured in complete Dulbecco’s Modified Eagle’s Medium (DMEM) containing 10% FBS, 2 mM glutamine, and 100 IU/ml penicillin-streptomycin. Cells were maintained in a humidified incubator with 5% CO_2_ at 37°C. For eCIRP treatment, PerM and iBMDMs were seeded into plates and cultured overnight. The media were changed to Opti-MEM medium (Cat# 31985-070; Thermo Fisher Scientific, Waltham, MA) 2 hours prior to treatment, and rmCIRP was applied at doses of 0.2 and 1.0 µg/ml in the experiments.

### PCR array and relative quantitative RT PCR analysis

Cellular RNA extraction was performed using the RNAspin mini-isolation kit (Cat# 25050072; Cytiva, Buckinghamshire, UK). For PCR Array analysis, cDNA was synthesized utilizing the RT first Strand kit (Cat# 330401; Qiagen, Germantown, MD). The impact of eCIRP on a panel of circadian genes was assessed through the mouse circadian rhythms RT2 Profiler PCR Array (Cat# 330231 PAMM-153ZA; Qiagen), comprising 84 circadian-related genes. Data analysis was carried out using an online spreadsheet-based tool provided by Qiagen. In the case of relative quantitative RT-PCR, cDNA synthesis employed MLV reverse transcriptase (Cat# 28025013; Thermo Fisher Scientific). PCR reactions were executed in a final volume of 20 μl, containing 0.08 μM of each forward and reverse primer (**Table 1**), cDNA, water, and SYBR Green master mix (Cat# 4368708; Thermo Fisher Scientific). Amplification and analysis were performed using a StepOnePlus real-time PCR machine (Thermo Fisher Scientific). Mouse β-actin mRNA served as an internal control for amplification, and relative gene expression levels were determined using the ^ΔΔ^CT method. The mRNA expression was presented as fold change compared to the control group.

**Table 1:** Primer sequences.

Gene	Accession No.	Forward Primer	Reverse Primer
β-actin	NM_007393	CGTGAAAAGATGACCCAGATCA	TGGTACGACCAGAGGCATACAG
BMAL2	NM_172309	GAAGAGCTGTACCGTCCCTG	CACCACCCGACTCTTTCTCC
CRY1	NM_007771	ATGTCCCGAGTTGTAGCAGC	GACTGTCCCCGTGAGCATAG
PER2	NM_011066	CTGCTTGTTCCAGGCTGTGGAT	CTTCTTGTGGATGGCGAGCATC
PD-L1	NM_021893	ACCAGCAGTCTGAGGGTCAA	CAATGAGGAACAACAGGATGG
IL-10	NM_010548	CAGAGCCACATGCTCCTAGA	GTCCAGCTGGTCCTTTGTTT
STAT3	NM_213659	AGTTCTCGTCCACCACCAAG	TAGCCAGACCCAGAAGGAGA
TREM-1	NM_021406	CTACAACCCGATCCCTACCC	AAACCAGGCTCTTGCTGAGA

### ELISA

Serum concentrations of eCIRP were assessed using an ELISA kit obtained from American Research Products (Cat# EM6504; Waltham, MA). The analysis of cytokine levels in cell culture supernatants, specifically TNF-α and IL-6, was conducted through ELISA kits procured from BD Biosciences (Cat# 558534 and 555240; San Jose, CA). All ELISA assays adhered to the protocols outlined by the respective manufacturers.

### Transfection of BMAL2 CRISPR activation plasmid

CRISPR Activation Plasmid products facilitate the identification and upregulation of specific genes through a D10A and N863A deactivated Cas9 (dCas9) nuclease fused to a VP64 activation domain, paired with sgRNA (MS2). This sgRNA is engineered to bind the MS2-P65-HSF1 fusion protein (27), forming a synergistic activation mediator (SAM) transcription activation system. SAM complex binds to a specific site upstream of the transcriptional start site (TSS) of the target gene, recruiting transcription factors and thereby activating endogenous transcription (27). The mouse BMAL2 CRISPR activation plasmid and the control CRISPR activation plasmid were procured from Santa Cruz Biotechnology (Cat# sc-435462-ACT and sc-437275; Dallas, TX). iBMDMs were seeded into 12-well plates in complete DMEM medium without antibiotics and cultured to 50-70% confluency. CRISPR BMAL2 activation and control plasmids were transfected into the cells using UltraCruz transfection reagents (Cat# sc-395739; Santa Cruz Biotechnology), followed by a 24-hour incubation period. Subsequently, cells were collected by centrifugation, and total RNA was extracted for the analysis of BMAL2 expression.

### Computational modeling

The promoter region of mouse CD274 (PD-L1) was identified by analyzing the 2000-nucleotide sequence upstream from the transcription start site of CD274 (NC_000085.7). Additionally, the sequence was retrieved from the Eukaryotic Promoter Database with the Promoter ID: CD274_1 (28). Structural modeling of the CD274 promoter was carried out using the 3DNA tool (29). This tool employs all possible canonical and non-canonical base pair geometries, relying on sequence-specific base pair step and base pair rigid body parameters, as well as template coordinates to generate a 3-dimensional structure model. The BMAL2 structure was modeled using the ITasser (Iterative Threading ASSEmbly Refinement) tool (30), which utilizes a threading approach to identify templates and model structures based on maximum percentage identity, sequence coverage, and confidence. The protein structure underwent further refinement through repeated relaxations, employing short molecular dynamics simulations for both mild (0.6 ps) and aggressive (0.8 ps) relaxations with a 4-fs time step after structural perturbations. Model refinement resulted in improved parameters, including enhanced Rama-favored residues and a decrease in poor rotamers. Additionally, a model for the scramble DNA sequence was generated.

The E-box motif of the BMAL2 transcription factor was identified within the CD274 (PD-L1) promoter. Subsequently, the BMAL2 protein was docked onto both the CD274 promoter and a scramble DNA sequence using the HDock tool (31) to create protein-DNA complexes. HDock employs a Fast Fourier Transform (FFT) based translational search algorithm, optimized through iterative knowledge-based scoring functions. Interactions between BMAL2 and the CD274 promoter, as well as the scramble DNA, were computed using the PDBePISA tool (32). To facilitate structure visualization, the BMAL2-CD274 promoter complex was rendered using PyMOL and Chimera tools (33).

### Surface plasmon resonance

To examine the direct interaction between recombinant human BMAL2 and PD-L1 promoter, surface plasmon resonance (SPR), OpenSPR (Nicoya, kitchener, Ontario), was performed between BMAL2 and PD-L1 promoter. Recombinant human BMAL2 protein (Cat# H00056938-Q01-25 µg; Novus Biologicals, Centennial, CO) was immobilized on high-sensitivity carboxyl sensor and the DNA oligo with the predicted sequence of BMAL binding site was injected as an analyte at concentration of 50 nM, 100 nM and 200 nM. Briefly, the carboxyl sensor was first cleaned by injection 150 µl of 10 mM HCL, followed by injection of 150 µl of the mixture of 1 aliquot of EDC and 1 aliquot of NHS to activate the sensor surface. An aliquot of 200 µl of 50 µg/ml of the ligand diluted in 10 mM sodium acetate (pH 5.5) was injected at 20 µL/min into flow cell-channel-2 of the sensor for immobilization. The flow cell channel-1 was used as a control to evaluate nonspecific binding. The binding analysis were performed at a flow rate of 30 μl per minute at 20°C. To evaluate the binding, the analyte was injected into flow cells-channel-1 and channel-2, and the real-time interaction data were analyzed by TraceDrawer software (Nicoya). The signals from the control channel (channel-1) were subtracted from the channel coated with the ligand (channel-2) for all samples. The data were globally fitted for 1:1 binding.

### Statistical analysis

Data are presented as mean ± SEM. Statistical comparisons among multiple groups were conducted using one-way ANOVA followed by Tukey’s test. For comparisons between two groups, an unpaired two-tailed Student’s t-test was employed. A significance level of P<0.05 was considered statistically significant.

## Results

### eCIRP induces macrophage endotoxin tolerance

**Figure 1:**
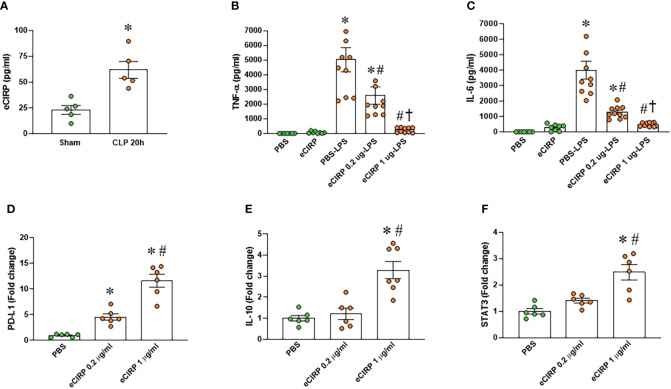
eCIRP promotes endotoxin tolerance in macrophages. **(A)** Plasma was collected from WT mice at 20 h after CLP to determine the circulating eCIRP levels. Data were presented as mean ± SEM (n=5/group). The groups were compared by unpaired two tailed Student’s t-test. *P < 0.05 vs. sham group. **(B, C)** Peritoneal macrophages (PerM) were isolated from WT male mice and pre-treated with rmCIRP (eCIRP; 0.2 and 1 µg/ml) for 24 h. The cells were then stimulated with LPS (50 ng/ml) for 5 h. The levels of **(B)** TNF-α and **(C)** IL-6 in the culture supernatants were measured by ELISA. The experiment was performed two times. Data presented were from two independent experiments and are expressed as the mean ± SEM (n=7-10/group). The groups were compared by One-way ANOVA and Tukey’s test. *P<0.05 vs. PBS, ^#^P<0.05 vs. PBS-LPS, ^†^P<0.05 vs. eCIRP 0.2 µg-LPS. **(D–F)** PerM were isolated from WT male mice and treated with 0.2 and 1 µg/ml eCIRP for 24 h. The expression of **(D)** PD-L1, **(E)** IL-10 and **(F)** STAT3 were analyzed by qPCR. The experiments were performed two times and the data were presented as mean ± SEM (n=6-7/group). The groups were compared by One-way ANOVA and Tukey’s test. *P<0.05 vs. PBS control, ^#^P<0.05 vs. eCIRP 0.2 µg/ml.

We examined the serum levels of eCIRP in septic mice and observed a significant elevation 20 hours after CLP compared to sham mice (**Figure 1A**). To investigate the induction of macrophage endotoxin tolerance by eCIRP, we pre-treated macrophages with varying doses of eCIRP for 24 hours. Subsequently, the media was replaced, and the cells were stimulated with LPS for 5 hours. Following this, the levels of TNF-α and IL-6 in the culture supernatants were evaluated. Notably, macrophages pre-treated with eCIRP exhibited a decreased release of TNF-α and IL-6 upon LPS challenge compared to cells pre-treated with PBS (**Figures 1B, C**). This observation suggests that pre-treatment with eCIRP induced macrophages to develop tolerance to LPS responsiveness. Interestingly, the magnitude of macrophage LPS tolerance was more pronounced in cells pre-treated with an increased dose of eCIRP compared to those treated with a lower dose. This implies a dose-dependent relationship, where the intensity of macrophage LPS tolerance correlates with the escalating doses of eCIRP. Higher doses of eCIRP resulted in more intense tolerance. Subsequently, we assessed the expression of genes commonly examined as markers of immune suppression in macrophages, including PD-L1, IL-10, and signal transducer and activator of transcription 3 (STAT3). Our data revealed a significant, dose-dependent increase in the expression of these genes upon eCIRP treatment (**Figures 1D–F**). These findings suggest that eCIRP promotes macrophage endotoxin tolerance.

### TREM-1 deficiency prevents eCIRP-induced macrophage endotoxin tolerance

**Figure 2:**
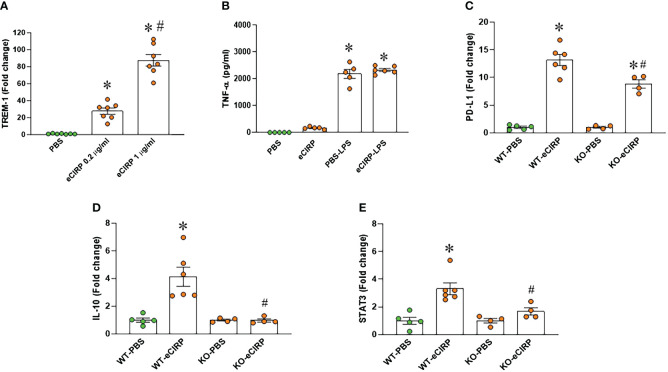
TREM-1 deficiency corrects endotoxin tolerance and restore the expression of its related genes expression in macrophages. **(A)** PerM were isolated from WT male mice and treated with eCIRP (0.2 and 1 µg/ml) for 24 h. The expression of TREM-1 was analyzed by qPCR. The experiments were performed twice, and the data were presented as mean ± SEM (n=7/group). The groups were compared by One-way ANOVA and Tukey’s test *P<0.05 vs. PBS, ^#^P<0.05 vs. eCIRP 0.2 µg/ml. **(B)** Murine peritoneal macrophages (PerM) were isolated from TREM-1^-/-^ male mice and pre-treated with rmCIRP (eCIRP; 1 µg/ml) for 24 h. The cells were stimulated with LPS (50 ng/ml) for 5 h. The release of TNF-α in the culture supernatants were assessed. Data are expressed as mean ± SEM (n=5-6/group). The groups were compared by One-way ANOVA and Tukey’s test. *P<0.05 vs. PBS. **(C–E)** PerM were isolated from WT and TREM-1^-/-^ male mice and treated with eCIRP (1 µg/ml) for 24 h. The gene expression of **(C)** PD-L1 **(D)** IL-10 and **(E)** STAT3 were analyzed by qPCR. The data were presented as mean ± SEM (n=4-6/group). The groups were compared by One-way ANOVA and Tukey’s test. *P<0.05 vs. PBS respectively, ^#^P<0.05 vs. WT eCIRP.

Given the recognized role of TREM-1 as a receptor for eCIRP-mediated function (12), we assessed TREM-1 expression in macrophages after eCIRP treatment. Our findings demonstrated a substantial increase in TREM-1 expression in macrophages treated with eCIRP compared to controls (**Figure 2A**). The involvement of TREM-1 in eCIRP-mediated immune tolerance remains elusive. To address this, we pre-treated TREM-1 knockout macrophages with either PBS or eCIRP for 24 hours. Subsequently, the media were replaced, and the cells were subjected to LPS stimulation for 5 hours. The culture supernatants were then collected to evaluate TNF-α release. Strikingly, our data revealed that TREM-1 knockout macrophages pre-treated with eCIRP did not exhibit any noticeable decrease in TNF-α release compared to PBS pre-treated cells (**Figure 2B**). This suggests that the deficiency of TREM-1 could attenuate macrophage endotoxin tolerance. Additionally, we identified that WT macrophages treated with eCIRP exhibited a significant increase in the expression of immune suppression markers, including PD-L1, IL-10, and STAT3 (**Figures 2C–E**). Intriguingly, the expression of these genes was significantly reduced in TREM-1 knockout macrophages compared to eCIRP-treated WT macrophages (**Figures 2C–E**). These findings imply that eCIRP induces macrophage endotoxin tolerance through TREM-1, highlighting TREM-1 as a crucial mediator in eCIRP-induced macrophage endotoxin tolerance.

### eCIRP increases BMAL2 expression in macrophages via TREM-1

**Figure 3:**
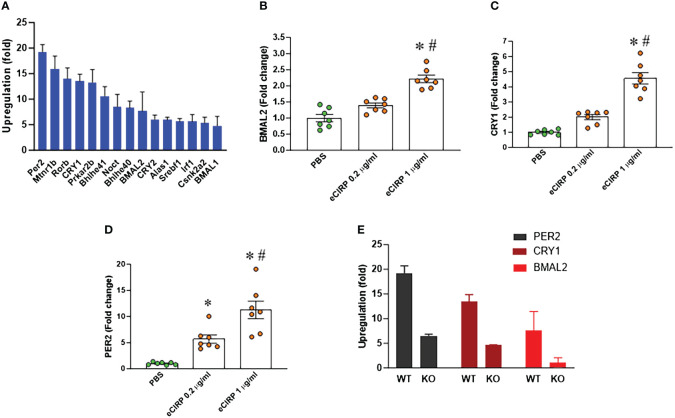
eCIRP increases BMAL2 expression via TREM-1 in macrophages. **(A)** Peritoneal macrophages (PerM) were isolated from WT male mice and treated with PBS or rmCIRP (eCIRP; 1 µg/ml) for 24 h. The fold increase of the expression of circadian genes was analyzed by PCR array. The data presented are from three independent experiments. **(B–D)** PerM were isolated from WT male mice and treated with eCIRP (0.2 and 1 µg/ml) for 24 h. The expression of **(B)** BMAL2, **(C)** CRY1, and **(D)** PER2 was analyzed by qPCR. The experiments were performed twice, and the data were presented as mean ± SEM (n=7/group). The groups were compared by One-way ANOVA and Tukey’s test *P<0.05 vs. PBS, ^#^P<0.05 vs. eCIRP 0.2 µg/ml. **(E)** PerM were isolated from TREM-1^-/-^ male mice and treated with PBS or eCIRP 1 µg/ml for 24 h. The fold increase of the expression of BMAL2, CRY1 and PER2 was obtained by PCR array and compared with WT PerM. The data presented are from three independent experiments.

We conducted a PCR array screening of circadian clock genes in peritoneal macrophages treated with eCIRP. The analysis indicated an augmentation in the expression of circadian genes. Specifically, the core circadian genes, including BMAL2, CRY1, and PER2, exhibited a marked upregulation in peritoneal macrophages treated with eCIRP (**Figure 3A**). Notably, the expression of another BAML isotype, BMAL1, was less increased in these cells, prompting our focus on BMAL2 and its role in eCIRP-induced macrophage immune tolerance. Validation through qPCR confirmed a significant upregulation of these genes (BMAL2, CRY1, and PER2) in peritoneal macrophages stimulated with eCIRP compared to controls (**Figures 3B–D**). Further investigation involved evaluating the expression of circadian genes (BMAL2, CRY1, and PER2) in both WT and TREM-1 knock-out macrophages treated with eCIRP. Interestingly, the expression levels of BMAL2, CRY1, and PER2 in eCIRP-treated macrophages lacking TREM-1 were notably lower than those in eCIRP-treated WT macrophages (**Figure 3E**). These results indicate that eCIRP, increased in sepsis, enhances the expression of circadian genes through the mediation of TREM-1.

### BMAL2 is a critical positive regulator of macrophage endotoxin tolerance

**Figure 4:**
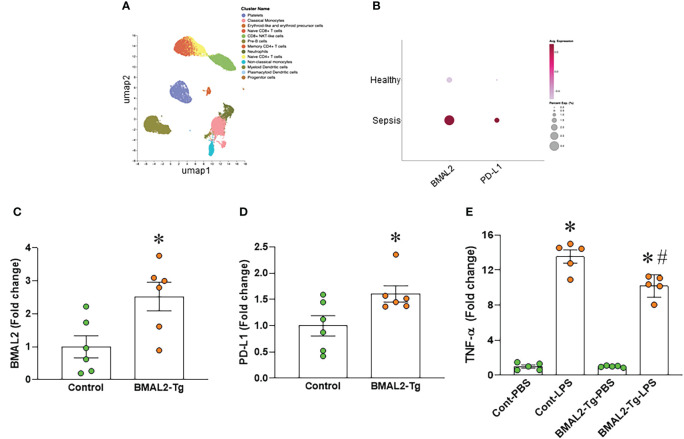
BMAL2 upregulates PD-L1 and promotes endotoxin tolerance in macrophages. **(A, B)** Human scRNA-seq data of healthy control (HC) and septic patients obtained from the GEO database (GSE167363) and analyzed by Cellenics are shown. **(A)** UMAP showing the unsupervised clustering of cells and **(B)** dot plots showing the gene expression of ARNTL2 (BMAL2) and CD274 (PD-L1) in the monocytes of HC and septic patients. **(C, D)** Immortalized bone marrow-derived macrophages (iBMDM) were transfected with BMAL2 expressing plasmid or non-specific control plasmid. After transfection, RNA was extracted from cells and the expression of **(C)** BMAL2 and **(D)** PD-L1 was determined by qPCR. The experiment was repeated. Data presented were from two independent experiments and expressed as the mean ± SEM (n=6/group). The groups were compared by unpaired two tailed Student’s t-test. *P<0.05 vs. control transfection group. **(E)** iBMDM were transfected with BMAL2 expression plasmid or non-specific control plasmid. After transfection, cells were stimulated with LPS (50 ng/ml) for 5 h. The release of TNF-α in the culture supernatants was assessed. PBS control groups were normalized as 1. Data are expressed as the mean ± SEM (n=5/group). The groups were compared by One-way ANOVA and Tukey’s test. *P<0.05 vs. PBS control, and ^#^P<0.05 vs. control transfection-LPS.

We analyzed the human scRNA-seq data of healthy control (HC) and septic patients obtained from the GEO database (GSE167363). We found that the gene expression of ARNTL2 (BMAL2) in the monocytes of septic patients was markedly higher than healthy controls (**Figures 4A, B**). Furthermore, we found that the gene expression of CD274 (PD-L1) in the monocytes of septic patients was markedly higher than healthy controls (**Figures 4A, B**). Having established that eCIRP induces both BMAL2 expression and macrophage endotoxin tolerance, we aimed to elucidate a potential positive correlation between them. To explore whether heightened BMAL2 expression as occur in eCIRP-treated condition could indeed enhance macrophage endotoxin tolerance, we activated BMAL2 expression in macrophages by transfecting them with either a control plasmid or an activation plasmid targeting BMAL2 expression. Following transfection, macrophages treated with the BMAL2 activation plasmid exhibited a significant increase in BMAL2 expression compared to those transfected with the control plasmid (**Figure 4C**). Subsequent treatment of these cells with LPS revealed that macrophages transfected with the BMAL2 activation plasmid displayed decreased TNF-α release and increased PD-L1 expression compared to control plasmid-treated cells (**Figures 4D, E**). These findings suggest that BMAL2 acts as a positive regulator of macrophage endotoxin tolerance.

### BMAL2 serves as an enhancer of PD-L1 expression by binding to the PD-L1 promoter

**Figure 5:**
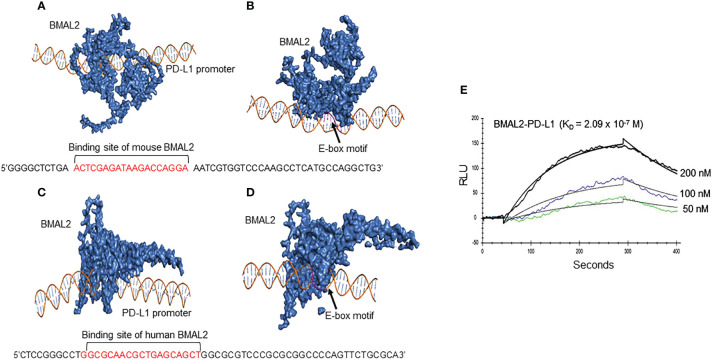
BMAL2 binds to the PD-L1 promoter to regulate PD-L1 expression. The binding sites between **(A)** mouse and **(C)** human BMAL2 with PD-L1 promoter sequences were predicted by 3D computational modeling. **(B, D)** Both mouse and human BMAL2 exhibit strong binding to the PD-L1 promoter’s E-box motif. **(E)** Biacore analysis with predicted putative DNA sequence from PD-L1 promoter as the analyte and BMAL2 as the ligand, showed strong binding between them with the binding affinity at *K_D_* of 2.09 × 10^−7^ M.

**Figure 6:**
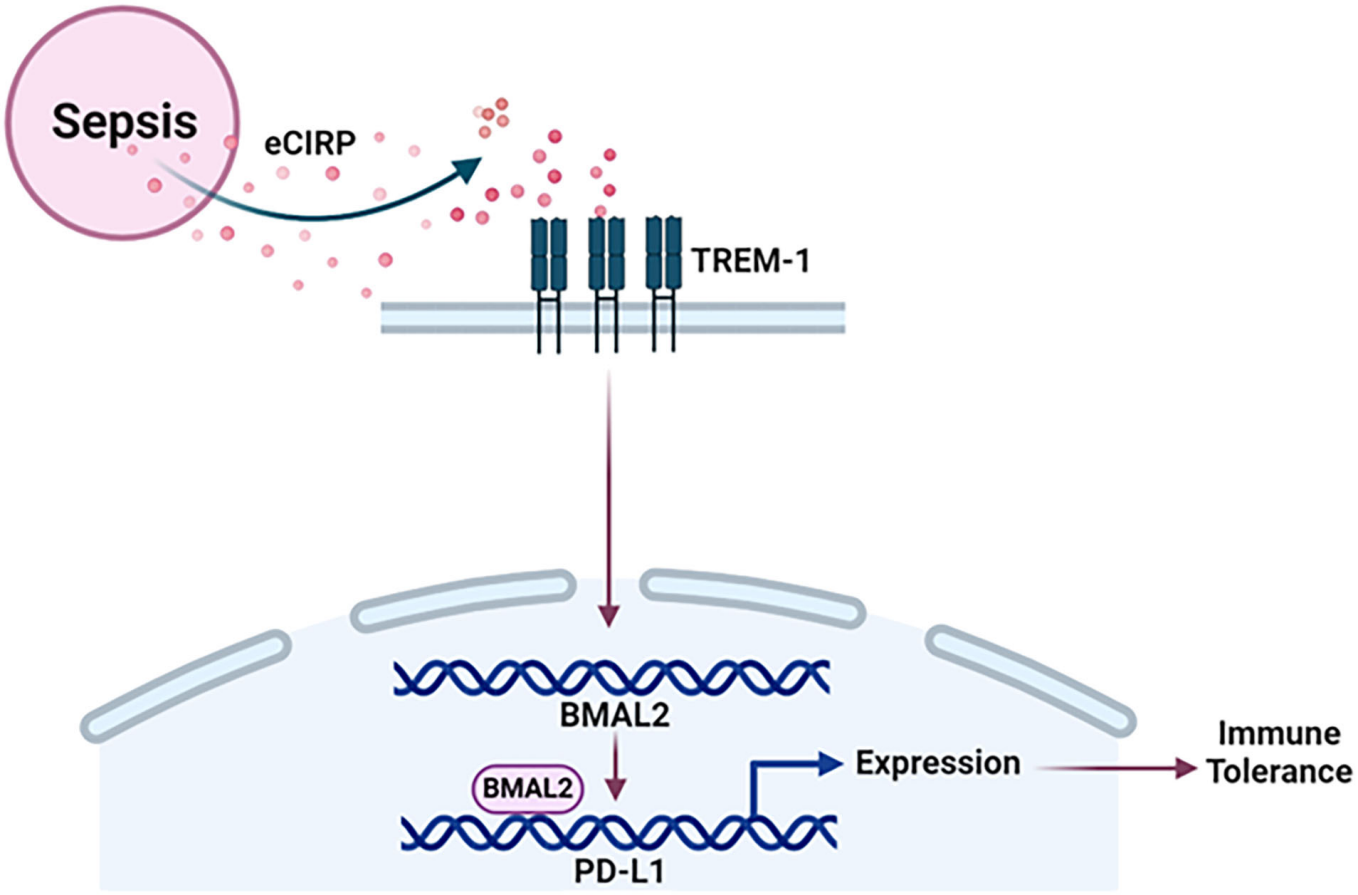
Summary of the findings. Sepsis causes the increased release of eCIRP to increase the expression of circadian gene BMAL2 through TREM-1. BMAL2 upregulates PD-L1 expression by binding to its promoter to induce macrophage endotoxin tolerance.

The promoter region of mouse CD274 (PD-L1) was identified by analyzing the 2000-nucleotide sequence upstream from the transcription start site of CD274 (NC_000085.7). Using *in silico* model we determined the binding of mouse and human BAML2 protein at the promoter region of mouse and human PD-L1 gene by the free energy of binding upon complex formation (Δ^i^G). Δ^i^G of BMAL2-PD-L1 promoter on mouse and human were -29.8 and -24.0 Kcal/mol, respectively (**Figures 5A, C**; **Table 2**). In comparison with BMAL1, BMAL2 showed stronger binding to PD-L1 promoter by the decrease in the free energy of binding upon complex formation, Δ^i^G (**Table 3**). Similarly, we also found that the mouse and human BMAL2 could bind to the E-box motif on the PD-L1 promoter with the free energy of binding upon complex formation Δ^i^G at -23.6 Kcal/mol in mouse and 14.9 Kcal/mol in human, respectively (**Figures 5B, D**; **Table 2**). We also experimentally validated the binding of human BMAL2 and human putative promoter sequence of PD-L1 by BIAcore assay revealing a strong binding between these two molecules at a *K_D_* value of 2.09 x 10^-7^ M (**Figure 5E**). These data reveal that BMAL2 positively regulate PD-L1 expression by binding to the PD-L1 promoter. Taken together, eCIRP increases BMAL2 expression in macrophages via TREM-1, ultimately leading to endotoxin tolerance (**Figure 6**).

**Table 2:** Binding of mouse and human BMAL2 on mouse and human PD-L1 promoter.

BMAL2-PD-L1 promoter	BMAL2 binding site on promoter	Binding Energy Δ^i^G (Kcal/mol)	E-box motif on promoter	Binding Energy Δ^i^G (Kcal/mol)
Mouse	5’ACTCGAGATAAGACCAGGA3’	-29.8	CAGGTG	-23.6
Human	5’GGCGCAACGCTGAGCAGCT3’	-24.0	CAGGTG	-14.9

The binding of mouse and human BMAL2 with PD-L1 promoter were predicted by 3D computational modeling. Both mouse and human BMAL2 exhibit binding to a DNA sequence on PD-L1 promoter and E-box. The interaction is expressed by free energy of binding (ΔiG) between BMAL2-PD-L1 promoter.

**Table 3:** The comparison between BMAL1 and BMAL2 on their binding to PD-L1 promoter.

Mouse	BMAL binding site on promoter	Binding Energy ∆^i^G (Kcal/mol)	E-box motifon promoter	Binding Energy ∆^i^G (Kcal/mol)
BMAL1-PD-L1 promoter	5’ACTCGAGATAAGACCAGGA3’	-23.07	CAGGTG	-23.4
BMAL2-PD-L1 promoter	5’ACTCGAGATAAGACCAGGA3’	-29.8	CAGGTG	-23.6
Human	BMAL binding site on promoter	Binding Energy ∆^i^G (Kcal/mol)	E-box motif on promoter	Binding Energy ∆^i^G (Kcal/mol)
BMAL1-PD-L1 promoter	5’GGCGCAACGCTGAGCAGCT3’	-21.0	CAGGTG	-15.8
BMAL2-PD-L1 promoter	5’GGCGCAACGCTGAGCAGCT3’	-24.0	CAGGTG	-14.9

The binding of BMAL1/BMAL2 with PD-L1 promoter were predicted by 3D computational modeling. Both mouse and human BMAL1 and BMAL2 can bind to specific DNA sequences on PD-L1 promoter and E-box. The binding is expressed as free energy of binding (Δ^i^G) between BMAL and PD-L1 promoter.

## Discussion

In this study, we have demonstrated that sepsis causes the increased release of eCIRP, leading to the promotion of endotoxin tolerance in macrophages. This process involves eCIRP’s ability to upregulate the expression of BMAL2 through the TREM-1 in macrophages. Subsequently, BMAL2 acts as a transcription factor, binding to the promoter of the PD-L1 gene. This binding results in an increased expression of PD-L1 in macrophages resulting immune tolerance (**Figure 6**). Immune tolerance refers to the immune system’s state of unresponsiveness to stimuli, which can contribute to the secondary infections, escalating morbidity, and mortality in various disease conditions, such as sepsis (34–36). The effects and underlying mechanisms of DAMPs in macrophage tolerance remain less elucidated. In our recent study, we identified a novel DAMP, eCIRP, and its involvement in immune suppression by inducing macrophage tolerance (34). In the current study, we unveiled a new mechanism of eCIRP-induced macrophage endotoxin tolerance. Our findings indicate that eCIRP treatment enhances the expression of the core circadian gene BMAL2 and its downstream molecules, CRY1 and PER2. Significantly, we have identified a correlation between elevated BMAL2 expression and the development of macrophage endotoxin tolerance. This association is intricately regulated by eCIRP and its receptor, TREM-1. Through inducible overexpression of BMAL2, we demonstrated that macrophages become more susceptible to endotoxin tolerance. Further investigations revealed that tolerant macrophages exhibit elevated expression of PD-L1, along with other surrogate markers of immune suppression. Importantly, we identified, for the first time, that BMAL2 positively regulates the expression of PD-L1 by binding to its promoter, enhancing its expression. These finding sheds light on the pathophysiology of eCIRP by utilizing the circadian gene BMAL2 in macrophage endotoxin tolerance. This novel mechanism provides valuable insights into potential therapeutic targets for mitigating immune suppression and improving outcomes associated with prolonged immune tolerance.

BMAL genes, namely BMAL1 and BMAL2, act as upstream regulators of the period (PER1, PER2, and PER3) and cryptochrome (CRY1, CRY2) genes (37). Functioning as transcription factors, either BMAL1 or BMAL2 forms a heterodimer with CLOCK, binding to E-box enhancer elements situated upstream of the PER and CRY gene promoters, thereby activating their transcription. The resultant PER and CRY proteins heterodimerize and initiate a feedback loop, repressing their own transcription by interacting with CLOCK/BMAL complexes (18, 19, 37). Given the pivotal role of BMAL in the regulation of PER and CRY genes as a primary transcription factor, our investigation delved into BMAL’s influence on the regulation of PD-L1, a key player in immune tolerance. Remarkably, following eCIRP treatment in macrophages, BMAL2 expression exhibited a significant increase compared to BMAL1. We identified an E-box element and a pertinent putative responsive element for BMAL2 binding on the PD-L1 promoter, positively modulating PD-L1 expression in macrophages. This discovery highlights the significance of spotlighting BMAL2 in our study, despite the relatively higher expression of PER2 compared to BMAL2 in eCIRP-treated macrophages. Nonetheless, future investigations regarding the impact of PER proteins on macrophage immune tolerance and the expression of their associated regulators would be of interest. Our data elucidate the pivotal role of BMAL2 and address the critical question of how DAMPs induce macrophage endotoxin tolerance in the context of sepsis. Notably, a recent study suggests that BMAL2 actively participates in circadian transcription, contributing to a broad spectrum of functions (23). Indeed, like our discovery of eCIRP-induced circadian clock gene expression, another study has highlighted the crucial involvement of intracellular CIRP in regulating circadian gene expression (38). Their findings demonstrated that loss-of-function experiments underscored the necessity of CIRP for maintaining high-amplitude circadian gene expression (38). It is noteworthy that while their study delved into the specific role of intracellular CIRP, our research centered on elucidating the influence of eCIRP on circadian gene expression in the context of sepsis. These collective insights highlight the importance of BMAL2 in the intricate interplay between circadian rhythms and immune responses, shedding light on a previously overlooked aspect of the molecular clock machinery and its implications in sepsis pathogenesis.

In an animal model of sepsis, the mortality rate is profoundly influenced by the timing of sepsis induction, emphasizing the pivotal role of the circadian system (39, 40). Notably, studies have elucidated the disrupted oscillation of circadian clock genes in mice after sepsis (41). The 24-hour circadian clock emerges as a vital regulator of macrophage immune function homeostasis (42). Recent studies have reported both protective and detrimental outcomes of BMAL1, notably utilizing BMAL1 knockout mice to support their findings. Peritoneal macrophages exhibit increased bacterial engulfment during the active phase (ZT12), when lower levels of BMAL are found, indicating that higher BMAL levels at the resting phase (ZT0) negatively regulate phagocytic function (42). Furthermore, proinflammatory cytokine release is elevated during the active phase (ZT12) post-bacterial infection (42). BMAL1 deficiency in macrophages amplifies cytokine expression compared to WT macrophages after bacterial stimulation, signifying BMAL1’s negative regulation of macrophage activation. BMAL1 deficiency in macrophages protects mice from pneumonia, exhibiting increased macrophage motility and bacterial clearance, resulting in a lower bacterial burden in blood (43). However, conditional deletion of BMAL1 in myeloid cells, disrupting the macrophage circadian clock, accelerates death in sepsis (14, 40). Global BMAL1 knockout mice exhibit significantly lower survival rates, displaying signs of premature aging and organ dysfunction (44). Recognizing the critical role of BMAL in survival and cellular function, our current study involved transfecting a BMAL2 activation plasmid to mimic increased BMAL2 expression post-eCIRP treatment. The heightened BMAL2 expression is associated with endotoxin tolerance, mirroring observations in eCIRP-treated macrophages, thereby emphasizing BMAL2’s crucial role in eCIRP-induced immune tolerance.

Immune checkpoints PD-1/PD-L1 serve as negative regulators of the immune system, modulating inflammation-associated immune suppression during sepsis (45). Macrophages express both PD-1 and PD-L1 receptors (46). The expression of PD-1/PD-L1 is associated with macrophage polarization from M1 to M2 subtype, altering their immune functions. PD-L1 has been shown to mediate M2 polarization, suppressing macrophage immune reactions after infection (7, 8, 46, 47). The interaction between PD-1 and PD-L1 has been demonstrated to reduce macrophage phagocytotic function (46, 47). In septic patients, peritoneal macrophages express high levels of PD-1, rendering these cells anergic and decreasing their bactericidal capacity (48). Conversely, PD-1^-/-^ mice exhibit a decrease in bacterial burden compared to wild-type mice during sepsis, leading to a higher survival rate (48). In our study, we identified that eCIRP induces circadian BMAL2 expression, promoting PD-L1 in macrophages. The interaction of PD-L1 with the PD-1 receptor on the cell surface inhibits macrophage activation. Building upon our previous study demonstrating that eCIRP induces M2 polarization and drives macrophages into endotoxin tolerance, we have uncovered the BMAL2-PD-L1 mechanism linking to macrophage endotoxin tolerance. In fact, we identified a responsive element at the mouse and human PD-L1 promoter using a novel approach of computational modeling and validated the silico finding by a quantitative BIAcore tool. Our data could support the future perspective that targeting PD-L1 in macrophages, achievable by addressing eCIRP and the BMAL2-mediated pathway, opens a novel therapeutic avenue for reversing endotoxin tolerance.

In our previous study, we demonstrated that eCIRP induces macrophage endotoxin tolerance via IL-6R-mediated STAT3 activation (34). Similarly, IL-6 treatment increased the activation of STAT3, a downstream mediator of tolerance. Since blocking IL-6R partially corrected endotoxin tolerance, this indicated the involvement of other pathways in eCIRP-induced macrophage endotoxin tolerance (34). In the current study, we elucidated a novel mechanism of macrophage endotoxin tolerance mediated via the TREM-1/BMAL2/PD-L1 axis. Studies have shown that the TREM-1 pathway is critical for eCIRP-induced IL-6 expression in macrophages (12), suggesting a potential link between the TREM-1 and IL-6-IL-6R-STAT3 axes in macrophage endotoxin tolerance. However, beyond this speculative connection, we have delineated the major role of eCIRP in upregulating the circadian gene BMAL2, which serves as a positive regulator of PD-L1 expression and contributes to macrophage endotoxin tolerance. Future studies to investigate the direct link between TREM-1 and the IL-6R-STAT3 axis in inducing macrophage endotoxin tolerance would be of great interest. To determine eCIRP’s impact on the expression of genes that serve as positive regulators or markers of immune tolerance, we stimulated macrophages with eCIRP and assessed gene expression at a single time point. This time point was based on findings from our previous research, which identified the optimal timing for these measurements (34). Specifically, our earlier study determined that stimulating macrophages with eCIRP for 24 hours followed by a 5-hour LPS treatment effectively induces endotoxin tolerance (34). In the present study, we found that eCIRP-induced IL-10, rather than PD-L1, exhibits complete inhibition in TREM-1 KO cells. One possible explanation for these results could be the involvement of non-TREM-1 receptors in eCIRP-induced PD-L1 expression. This suggests the need for future investigation into whether IL-10 is a clock-related gene and whether BMAL2 knock-in can enhance IL-10 expression. Additionally, the optimal induction or inhibition of different gene expressions may depend on various factors, including timing, stimulation dose, and the complex interplay of several signaling pathways, which can influence the outcomes of gene expression and regulation.

In this study, we have unveiled a novel mechanism wherein eCIRP induces macrophage endotoxin tolerance through TREM-1-mediated upregulation of BMAL2. Our focus extends to prospective investigations involving neutrophils, recognizing these innate immune cells as susceptible to tolerance, and exploring potential implications in adaptive immune cells such as lymphocytes. These findings not only contribute to our understanding of immune response regulation but also pave the way for future endeavors. Directing interventions towards the eCIRP and BMAL2-mediated pathway emerges as a promising therapeutic approach to reverse immune tolerance.

## Data availability statement

The datasets presented in this study can be found in online repositories. The names of the repository/repositories and accession number(s) can be found below: GSE266607 (GEO).

## Ethics statement

The animal study was approved by Feinstein Institutes for Medical Research. The study was conducted in accordance with the local legislation and institutional requirements.

## Author contributions

MZ: Writing – review & editing, Writing – original draft, Visualization, Validation, Resources, Methodology, Investigation, Formal analysis, Data curation, Conceptualization. MA: Funding acquisition, Software, Writing – review & editing, Writing – original draft, Visualization, Validation, Supervision, Resources, Project administration, Methodology, Investigation, Formal analysis, Data curation, Conceptualization. JL: Writing – review & editing, Methodology, Formal analysis, Data curation. AJ: Writing – review & editing, Methodology, Investigation, Formal analysis. GM: Writing – review & editing, Methodology, Investigation, Formal analysis. AM: Writing – review & editing, Software, Methodology, Investigation. PW: Writing – original draft, Writing – review & editing, Validation, Supervision, Resources, Project administration, Investigation, Funding acquisition, Formal analysis, Conceptualization.
